# Single Amino Acid Residue W33 of *tva* Receptor Is Critical for Viral Entry and High-Affinity Binding of Avian Leukosis Virus Subgroup K

**DOI:** 10.3390/v17050709

**Published:** 2025-05-15

**Authors:** Eliška Gáliková, David Přikryl, Salomé Prost, Dana Kučerová, Kateřina Trejbalová, Jiří Hejnar

**Affiliations:** Laboratory of Viral and Cellular Genetics, Institute of Molecular Genetics, The Czech Academy of Sciences, CZ-14220 Prague, Czech Republic; galikova@img.cas.cz (E.G.); prikryl.david@gmail.com (D.P.); salome.prost@img.cas.cz (S.P.); dana.kucerova@img.cas.cz (D.K.); katerina.trejbalova@img.cas.cz (K.T.)

**Keywords:** avian leukosis virus, *tva* receptor, chicken, guineafowl

## Abstract

Avian leukosis virus (ALV), the prototypical alpharetrovirus, causes tumorigenesis, immunosuppression, and wasting disease in poultry. The ALV genus is classified into ten subgroups, which differ in their host range, cell tropism, and receptor usage. The subgroups A, B, K, and J cause significant economic losses worldwide. The most recently discovered subgroup, ALV-K, which is now widespread in China, has been shown to use the *tva* cell receptor and share it with ALV-A. However, the specific amino acid residues crucial for ALV-K host cell entry remain unknown. Using precise *tva* expression and chimeric *tva* receptors, we further elucidated the significance of the cysteine-rich domain in mediating interactions with both ALV-A and ALV-K. Through a comprehensive analysis of mutated *tva* receptor variants, we pinpointed tryptophan at position 33 (W33) as a pivotal amino acid residue essential for ALV-K virus binding and entry. Of note is the finding that the substitution of W33 induced resistance to ALV-K while preserving sensitivity to ALV-A. This study not only represents an advance in the understanding of the specificity of the *tva* receptor for ALV-K, but also offers a biotechnological strategy for the prevention of ALV-K infections in poultry.

## 1. Introduction

Avian leukosis virus (ALV) is an alpharetrovirus linked to tumorigenesis, immunosuppression, and wasting disease in domestic poultry [1]. The ALV genus is classified into ten subgroups, which differ in their host range in chicken breeds and wild bird species, cell tropism, pathogenicity, and superinfection interference, whereby all these differences are determined by the specific receptor usage [2]. The enormous variability of ALV envelope glycoproteins corresponds with numerous resistant receptor variants, suggesting coevolution of virus and host by positive selection of critical amino acid residues. The exogenous subgroups A, B, K, and J cause significant economic losses in the poultry industry [3,4,5]. ALV-A infections have been reported worldwide while recently occurring ALV-K outbreaks have predominantly been reported from Eastern Asia [6]. JS11C1 is a prototypic strain of the newly established subgroup ALV-K first identified in the Chinese Luhua chicken [7]. In recent years, more ALV-K isolates have been found in chickens displaying various pathologies [4].

The JS11C1 isolate, as probably all other ALV-Ks, has been shown to enter cells through the *tva* receptor [8], a low-density lipoprotein receptor-related protein A, long-known as the receptor for ALV-A [9,10]. *Tva* is an orthologue of mammalian CD320 with its primary role of intracellular uptake of transcobalamin-bound vitamin B12 [11]. Although ALV-K envelope (env) sequences cluster separately from ALV-A, they share the same cellular receptor and ALV-A establishes a strong interference with ALV-K in superinfection experiments [8]. It remains to be determined, which epitope(s) the ALV-A and ALV-K env glycoproteins bind and which amino acid residues are critical for virus entry. Such knowledge has already been useful for the derivation of virus-resistant chicken lines by gene editing technology [12].

Partial or complete loss of *tva* was described in chicken breeds or galliform species. Inbred chicken line 7(2) is ALV-A-resistant due to a frame-shifting 4-bp insertion in *tva* exon 1, ultimately abolishing the expression of the *tva* receptor [13]. Quite common are *tva* alleles with deletions of various extents within the first intron. These deletions, found in both domestic chickens and jungle fowls, always include the branch point and, therefore, strongly reduce the correctly spliced transcript and *tva* display on the plasma membrane. In homozygous states, such *tva* alleles cause partial resistance to ALV-A [14,15]. Although these null mutations are not deleterious, knockout of *tva* conferred not only ALV-A resistance but also a disorder of vitamin B12 metabolism [16] in accordance with *tva*’s physiological function [11].

The extracellular part of the *tva* receptor contains a cysteine-rich domain (CRD) with three disulfide bridges that are crucial to the stability of the protein structure [17]. Furthermore, the acidic residues of the CRD domain can interact with positively charged residues found in hypervariable hr1 and hr2 regions of ALV-A (and ALV-K) but not of other subgroups, such as ALV-B to E env glycoproteins [18]. Tryptophan residue 48 (W48) within CRD was identified as a residue critical for ALV-A entry [17,19]. Moreover, another single amino acid substitution, C40W, also abrogates ALV-A entry and decreases virus-receptor binding affinity [13]. Very recently, four residues E53, L55, H59, and G70 were found to mediate ALV-K binding and entry [20]. However, none of these mutations were simultaneously tested for entry and infection with ALV-A. Therefore, gross structural changes generally abrogating the receptor function cannot be excluded. Differential resistance to one subgroup while retaining susceptibility to the other would point to residues that might be used for precise gene editing and derivation of single- or double-resistant chicken lines.

For the derivation of ALV-A and ALV-K-resistant chicken lines by gene editing technology, single amino acid deletions or substitutions of critical residues, which most probably do not affect the vitamin B12 receptor function, represent the best option. In order to find such mutations, we decided to analyze polymorphisms discriminating the susceptibility or resistance to ALV-A and ALV-K subgroups. The host ranges of both subgroups are similar and most inbred chicken lines or galliform species differ only quantitatively in their susceptibility to ALV-A and ALV-K. The only exception is helmeted guineafowl (*Numida meleagris*), which is resistant to ALV-K, but highly sensitive to ALV-A [8]. In this study, we employed the comparison of chicken (*Gallus gallus*; gg*tva*) and guineafowl *tva* (nm*tva*) to identify the subgroup-specific requirements for receptor binding at the level of single amino acids.

## 2. Materials and Methods

### 2.1. Cell Culture

All cells were cultured in a mixture of Dulbecco’s modified Eagle’s medium and F-12 medium (1:1 *v*/*v*; Sigma-Aldrich, Vienna, Austria) with penicillin-streptomycin (100 μg/mL each, Sigma-Aldrich, Vienna, Austria). The growth medium of the DF1 chicken fibroblast cell line, genetically modified DF1-Δ*tva* cells [21], *tva*-transduced DF1 cells, and embryo fibroblasts from helmeted guineafowl (*Numida meleagris*; see [8] for the source) was supplemented with 4.5% fetal calf serum, 4.5% calf serum, and 1% chicken serum. The growth medium of the HEK293T cell line was supplemented with 5% fetal calf serum and 5% calf serum. The cells were maintained in a controlled environment of 5% CO_2_ at 37 °C.

### 2.2. Replication-Competent Avian Retroviruses

Replication-competent retroviral vectors, namely ALV-based RCASBP(A)GFP and RCASBP(K)GFP were utilized for this study [8,14,22]. These vectors carried the *envelope* (*env*) gene of A and K, strain JS11C1, respectively, and the GFP fluorescent reporter protein to detect the infected cells. To produce the infectious virus, the respective plasmids were transfected into the chicken DF1 cells using Lipofectamine 3000 (Thermo Fisher Scientific, Waltham, MA, USA) according to the manufacturer’s protocol. Nine days post-transfection and after two cell passages on the second and sixth day, the medium containing virus particles was collected. The harvested medium was centrifuged at 2000× *g* for 10 min and the resulting supernatant filtered using a 0.45 μm filter. Aliquots were stored at −80 °C until further use. The virus titers were assessed by terminal dilution and infection of chicken DF1 cells. The detection of GFP-positive cells was performed by flow cytometry using the LSRII analyzer (BD Biosciences, Franklin Lakes, NJ, USA). Both retroviral vectors exhibited titers of 10^6^ infectious units (IU) per ml.

### 2.3. Construction, Production, and Transduction of tva Expression Vectors

Coding sequences for the avian receptors of domestic chicken (gg*tva*) and guineafowl (nm*tva*) were amplified by PCR using cDNAs prepared from chicken DF1 cells and guineafowl primary fibroblasts, respectively. The amplification introducing suboptimal starting codon TTG was carried out using primers gg/nm*tva*-TTG-FW and gg/nm*tva*-TTG-RV (Table 1). Chimeric receptors containing combinations of sequences originating from either gg*tva* or nm*tva* were produced by fusing amplified segments of the abovementioned receptors using primers Segment_A-FW and RV, Segment_B-FW and RV, and Segment_C-FW and RV (Table 1). Specific mutations in the gg*tva* receptor were introduced using the following primer pairs: gg*tva*-S12P-FW and RV, gg*tva*-H17Q-FW and RV, gg*tva*-Δ19-23aa-FW and RV, gg*tva*-T26S-FW and RV, gg*tva*-D27V-FW and RV, gg*tva*-Y29S-FW and RV, gg*tva*-D42S-FW and RV, gg*tva*-G44E-FW and RV, gg*tva*-W48A-FW and RV, gg*tva*-W48V-FW and RV, gg*tva*-W33A-FW and RV, and gg*tva*-W33V-FW and RV (Table 1). The *tva* coding sequences with the TTG start codon were fused with mCherry at its C terminus and inserted into the previously described pFuTraP plasmid [23] followed by IRES and iRFP713. All cloning steps were performed using the In-Fusion cloning kit (Takara Bio, San Jose, CA, USA). The resulting vectors were propagated in *E. coli* Stbl2 strain (NEB, Ipswitch, MA, USA) and their sequences were verified through Sanger sequencing.

To generate *tva* transducing viruses, DF1-Δ*tva* cells were cotransfected with pGag-pol [22], pVSV-G (Takara Bio, San Jose, CA, USA), and the respective variant of pFuTraP plasmid (ratio 0.3:0.2:0.5, respectively). pGag-pol encodes the structural viral proteins and enzymes whereas pVSV-G encodes the pantropic envelope glycoprotein of vesicular stomatitis virus. Three days post-transfection, the medium containing replication-defective viral particles was collected, centrifuged at 2000× *g* for 10 min, and filtered through a 0.45 μm filter. Stable ectopic expression of the chimeric receptors was achieved by transduction of these viral particles into DF1-Δ*tva* cells. Transduced cells were enriched for iRFP713 positivity by sorter BD Influx (BD Biosciences, Franklin Lakes, NJ, USA) after three passages.

### 2.4. Infection and Spinoculation Assay

Infections with RCASBP(A)GFP and RCASBP(K)GFP were conducted according to the following procedure: the day before infection, 5 × 10^4^ cells were seeded on a 24-well plate. The infection was carried out at a multiplicity of infection (MOI) of 1.

For spinoculation, the cells were seeded under the same conditions; 100 μL of virus supernatant and 200 μL of culture medium were added and the 24-well plates were subjected to centrifugation at 1200× *g* for 2 h at 25 °C. After centrifugation, an additional 200 μL of culture medium was added. Three days after infection or spinoculation, the MFI of GFP, mCherry, and iRFP713 was analyzed by flow cytometry using the LSRII analyzer (BD Biosciences, Franklin Lakes, NJ, USA)

### 2.5. Immunoadhesin

Cloning of a plasmid pCB6-SUK-rIgG, encoding the soluble SUK-rIgG, was performed using the pCB6-SUA-rIgG [22] digestion with *Sac*I and *Stu*I restriction enzymes, followed by insertion of a SUK PCR fragment. The PCR reaction used pRCASBP(K)GFP as a template and employed the following primers: forward (5′-GCATTTCTGACTGGATACCCTGGGGAGACAAGCAAGAAG-3′) and reverse (5′-CGGACGACTGGGAATTCCTTGCCATGCGCGAT-3′). Insertion was achieved using the In-Fusion cloning kit (TaKaRa) according to the manufacturer’s protocol. The constructed vector was propagated in E. Coli XLBlue and its sequence was validated by Sanger sequencing.

To produce the SUA-rIgG and SUK-rIgG immunoadhesins, HEK293T cells were transfected using Lipofectamine 3000 (Thermo Fisher Scientific, Waltham, MA, USA) according to the manufacturer’s protocol. Three days post-transfection, the supernatants were collected, filtered through the 0.45 μm filter, concentrated, aliquoted, and stored at −80 °C. The concentration of immunoadhesins was determined by serial dilution and ELISA as previously described for mouse IgG, with adaptations for rabbit IgG (rIgG) and the use of an anti-rabbit IgG detection system [24].

To assess the binding of SUA-rIgG and SUK-rIgG to the virus receptor on the cell surface, cells were trypsinized, washed in PBS, and 2.5 × 10^5^ cells were incubated with 250 μL of supernatant with approximately 30 ng of SUA-rIgG or SUK-rIgG at 4 °C for 1 h. After incubation, cells were washed three times with PBS supplemented with 4% calf serum. Subsequently, the cells were incubated for 30 min at 4 °C with anti-rabbit IgG conjugated to Alexa Fluor 488 antibody (Abcam, Cambridge, UK, 1:1000 dilution in PBS with 4% calf serum). After staining, cells were washed three times with PBS supplemented with 4% calf serum and stained with Hoechst 33258 solution (Sigma-Aldrich, Vienna, Austria). The intensity of Alexa Fluor 488-, mCherry-, and iRFP71 was analyzed by flow cytometry using the LSRII analyzer (BD Biosciences, Franklin Lakes, NJ, USA).

### 2.6. Flow Cytometry Analysis

The cells for flow cytometry analysis were washed with PBS, trypsinized, pelleted by centrifugation, and resuspended in Hoechst 33258 solution (Hoechst: PBS; 1:1000, Sigma-Aldrich, Vienna, Austria). FACS analysis was performed to quantify the MFI and percentage of GFP-, Alexa Fluor 488-, mCherry-, or iRFP713-positive cells. The analysis was carried out using the LSRII flow cytometer (BD Biosciences, Franklin Lakes, NJ, USA) with the following configuration: Hoechst 33258 using 405 nm laser and bandpass filter 450/50, GFP and Alexa Fluor 488 using 488 nm laser and bandpass filter 525/50, mCherry using 561 nm laser and bandpass filter 610/20, and iRFP713 using 637 nm laser and bandpass filter 705/20 (BD Biosciences, Franklin Lakes, NJ, USA). FlowJo 10.8 was used for the analysis of the detected signals.

### 2.7. Sensitivity Calculation

Cell sensitivity to virus infection was calculated using a formula: Sensitivity = −*ln* (1 − percentage fraction of GFP-positive cells). This approach has been described in references [23,25]. Furthermore, the sensitivity to RCASBP(A)GFP and RCASBP(K)GFP was normalized to the sensitivity of DF1-Δ*tva*-gg*tva*.

### 2.8. RNA Isolation, cDNA Synthesis, and qRT-PCR

Total RNA was isolated from cultured cells using RNAzol RT (Molecular Research Center Inc., Cincinnati, OH, USA). Two micrograms of RNA were reversely transcribed using Protoscript II reverse transcriptase (NEB, Ipswitch, MA, USA). The qPCR reaction included cDNA sample with MESA GREEN qPCR MasterMix Plus (Eurogentec, Liège, Belgium) and primers targeting *GAPDH* (5′CATCGTGCACCACCAACTG and 5′CGCTGGGATGATGTTCTGG) or *tva* (5′GGTTGTTGGAGCTGCTGGT and 5′CACTCCAGCGGGTAGCAGTC. The samples were run on a CFX96TM real-time instrument (BioRad, Hercules, CA, USA) with a three-step protocol (1 cycle of 8 min at 95 °C followed by 40 cycles of 15 s at 95 °C, 25 s at 60 °C, and 35 s at 72 °C. Cycles of quantification (Cq) values were generated by the CFX Manager software, and the specificity of the PCR products was confirmed by melting curves analysis.

### 2.9. Statistical Analysis

All assays were performed and carried out at least three times independently to achieve the purpose of three biological replicates. Prism software (Version 8) was used to perform two-way ANOVA tests to assess differences between groups. Statistical significance was set at *p* < 0.05.

## 3. Results

### 3.1. Experimental Design and Generation of Retroviral Vector for Ectopic tva Expression

**Figure 1:**
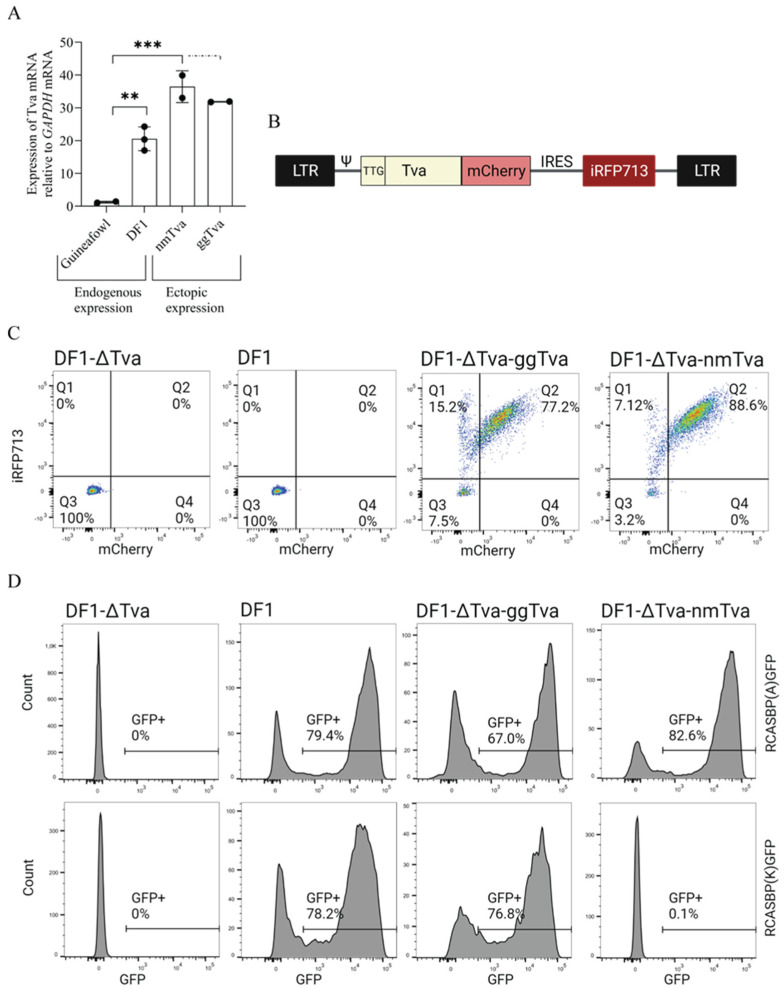
Experimental design and susceptibility of cells with ectopic expression of the *tva* receptor. (**A**) Endogenous *tva* mRNA expression relative to GAPDH gene in guineafowl embryo fibroblasts, chicken DF1 cells, ectopic expression of chicken (gg*tva*), and guineafowl (nm*tva*) *tva* receptors. Data are shown as means ± standard errors of three biological replicates, ** *p* < 0.01, *** *p* < 0.001. Statistical analysis was performed using a two-way ANOVA test. (**B**) Scheme of the pFuTrap vector used for ectopic expression of *tva* receptor. The vector contains two flanking long terminal repeats (LTR), packaging signal (ψ), suboptimal initiation codon TTG, *tva* receptor with mCherry fluorescence protein fused to its C-terminus followed by internal ribosomal entry site (IRES) and iRFP713 fluorescence protein. (**C**) The transduction efficiency was determined by iRFP713 fluorescence intensity and the *tva* expression was represented by mCherry fluorescence intensity. Δ*tva* and DF1 cells were negative controls. (**D**) The infection of transduced cells and control cells by subgroups A and K. (**C**,**D**) Representative data of triplicate experiments with average percentage of the fluorescence protein are shown.

In the past, we have described a chicken DF1 cell clone DF1-Δ*tva* (referred to as Δ*tva*) with a *tva* gene knockout, which is resistant to both ALV-A and ALV-K [21]. This cell clone is suitable for ectopic expression of *tva* variants combining the chicken and guineafowl sequences and analysis of the virus entry by reporter-transducing replication-competent ALV-A and ALV-K vectors. To establish stable ectopic expression of the *tva* receptor variants, we adapted a versatile avian replication-defective retroviral vector pFuTraP [23]. Because endogenous *tva* receptor protein(s) are expressed at very low levels in avian cells and its overexpression can erase differences in virus binding [13], we compared the ectopic *tva* protein levels to its endogenous levels (Figure 1A). Interestingly, the endogenous expression of *tva* in the guineafowl embryo fibroblasts was extremely low (Figure 1A), but this did not prevent the efficient ALV-A infection [8]. To match the ectopic expression of *tva* variants, we introduced a suboptimal TTG start codon into the *tva* gene and prepared the dual fluorescence pFuTraP-*tva* vectors encoding the modified *tva*-mCherry fusion protein variants followed by an IRES-iRFP713 cassette (Figure 1B). The iRFP713 fluorescence allowed precise control of transduction efficiency whereas mCherry served for quantification of *tva* receptor variants expression. We infected the DF1-Δ*tva* cells with pFuTraP-*tva*-transducing virus pseudotyped with VSV-G. Transduced cells were analyzed by fluorescence-activated cell sorting (FACS) according to iRFP713 and mCherry positivity, to verify the vast majority of cells were successfully expressing either chicken gg*tva* or guineafowl nm*tva* receptors (Figure 1C). The subsequent infection with replication-competent RCAS vectors of A or K subgroup specificity, the RCASBP(A)GFP or RCASBP(K)GFP, respectively, confirmed the resistance of *tva* receptor from guineafowl to ALV-K, while susceptibility to ALV-A was preserved (Figure 1D), which was observed previously [8].

### 3.2. Cysteine-Rich Domain of tva Is Important for Sensitivity to ALV-K

**Figure 2:**
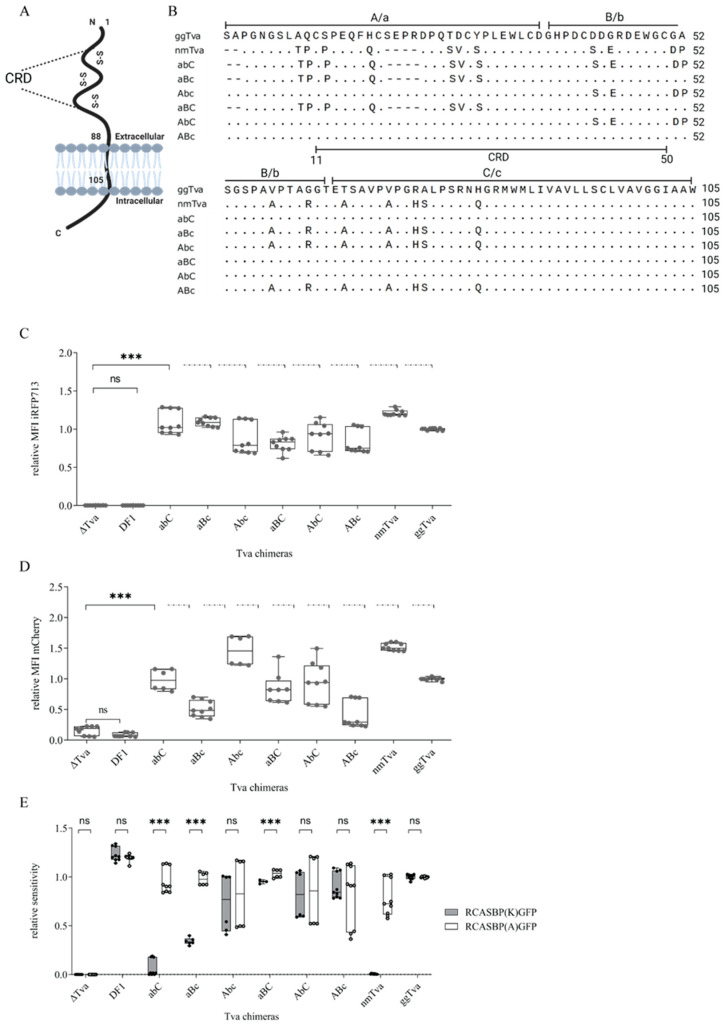
Mapping the extracellular segments of chicken and guineafowl *tva* receptor responsible for the resistance to ALV-K. (**A**) Schematic representation of *tva* receptor. (**B**) Amino acid alignment of the extracellular part of chicken (gg*tva*, Genbank No. 420066), guineafowl (nm*tva*, Genbank No. 110388860), and chimeric *tva* receptors. The signal peptide at the N terminus is not shown. Three segments of the extracellular part, spanning amino acids 1–36, 37–64, and 65–105, termed A, B, and C in gg*tva* and a, b, and c in nm*tva*, respectively, used for cloning chimeric *tva* receptors are shown as a section line over the sequence. All chimeric *tva* sequences designated as abC, aBc, Abc, aBC, AbC, and ABc are aligned at the bottom. (**C**) The transduction efficiency of the *tva*-expressing vector was determined as iRFP713 MFI normalized to the MFI of gg*tva*; Δ*tva* and DF1 were used as negative controls. (**D**) The relative expression of *tva* receptor variants fused with mCherry normalized to gg*tva* expression. (**E**) The sensitivity of cells expressing the chimeric *tva* variants to infection with RCASBP(A)GFP or RCASBP(K)GFP reporter viruses. The fraction of GFP-positive cells was normalized to the gg*tva*. Δ*tva* was used as a negative control, DF1 and gg*tva* were positive controls. Data are shown as means ± standard errors of three independent experiments with three biological replicates, *** *p* < 0.001. Statistical analysis was performed using a two-way ANOVA test. ns: non-significant.

Chicken *tva* is highly homologous to guineafowl *tva* in its extracellular part and, in particular, in all six conserved cysteine residues clustered in the cysteine-rich domain (CRD). These cysteines form disulfide bridges and maintain the correct structure of the LDL-A module of *tva* (Figure 2A) [26]. The alignment shows several single amino acid substitutions and two short deletions in the guineafowl *tva* (Figure 2B). To map the polymorphisms associated with the resistance of guineafowl *tva* to ALV-K, we divided the extracellular part of the guineafowl *tva* receptor into three segments spanning amino acids 1–36, 37–64, and 65–105, (termed a, b, and c, respectively), which were then swapped for the respective chicken *tva* segments (termed A, B, and C, respectively), either alone or in combination. All chimeric variants (dubbed as abC, aBc, Abc, aBC, AbC, and ABc and listed in alignment, Figure 2B) were introduced into the pFuTraP expression retroviral vector as mCherry-fusion coding sequences and transduced in DF1-Δ*tva* cells.

The iRFP713 mean fluorescence intensity (MFI) of the transduced cells showed efficient transduction of all FuTraP variants encoding chimeric *tva* as well as wild-type (wt) nm*tva* and gg*tva* (Figure 2C). Moreover, this was also documented at the protein levels defined by the mCherry MFI and normalized to FuTraP-gg*tva* cells. Despite variation between individual chimeric *tva* variants, we observed a maximum two-fold deviation from both nm*tva*- and gg*tva*-transduced DF-1-Δ*tva* cells, which verified the proper expression of our constructs (Figure 2D).

To determine the ability of respective chimeric *tva* receptors to discriminate between subgroups A and K, the cells were infected in parallel with the same amount of RCASBP(A)GFP or RCASBP(K)GFP reporter viruses. DF1 cells, DF1-Δ*tva* cells, and DF1- Δ*tva* cells expressing gg*tva* or nm*tva* were used as controls. Sensitivity to infection was determined as a fraction of GFP-positive cells and was normalized to the expression of the gg*tva*. When challenged with RCASBP(A)GFP, we did not observe any difference in infection in cells expressing single chimeric or wt *tva* receptors, indicating not only a sufficient level of expression but also a proper presentation of *tva* molecules at the plasma membrane. In striking contrast, the chimeric guineafowl *tva* variants containing the middle or last part of chicken *tva* (abC and aBc) were significantly less sensitive to RCASBP(K)GFP (Figure 2E). Interestingly, the combination of the middle and the last part of chicken *tva* induced no significant change in susceptibility to ALV-K. Ultimately, none of the a, b, or c segments of nm*tva* alone could confer the resistance to the K subgroup (Figure 2E). This leads to the conclusion that the a segment of nm*tva* is necessary but not sufficient to provide resistance to subgroup K alone, and a combination with the b or c (less effective) segments of nm*tva* is required. Although resistance to the ALV-K subset clearly maps to the central portion of the CRD *tva* bead, our results suggest that the polymorphisms present here interact along the entire CRD and resistance requires a combination of substituted amino acids.

### 3.3. Resistance of Guineafowl tva to ALV-K Depends on a Combination of Polymorphic Amino Acid Residues in CRD

**Figure 3:**
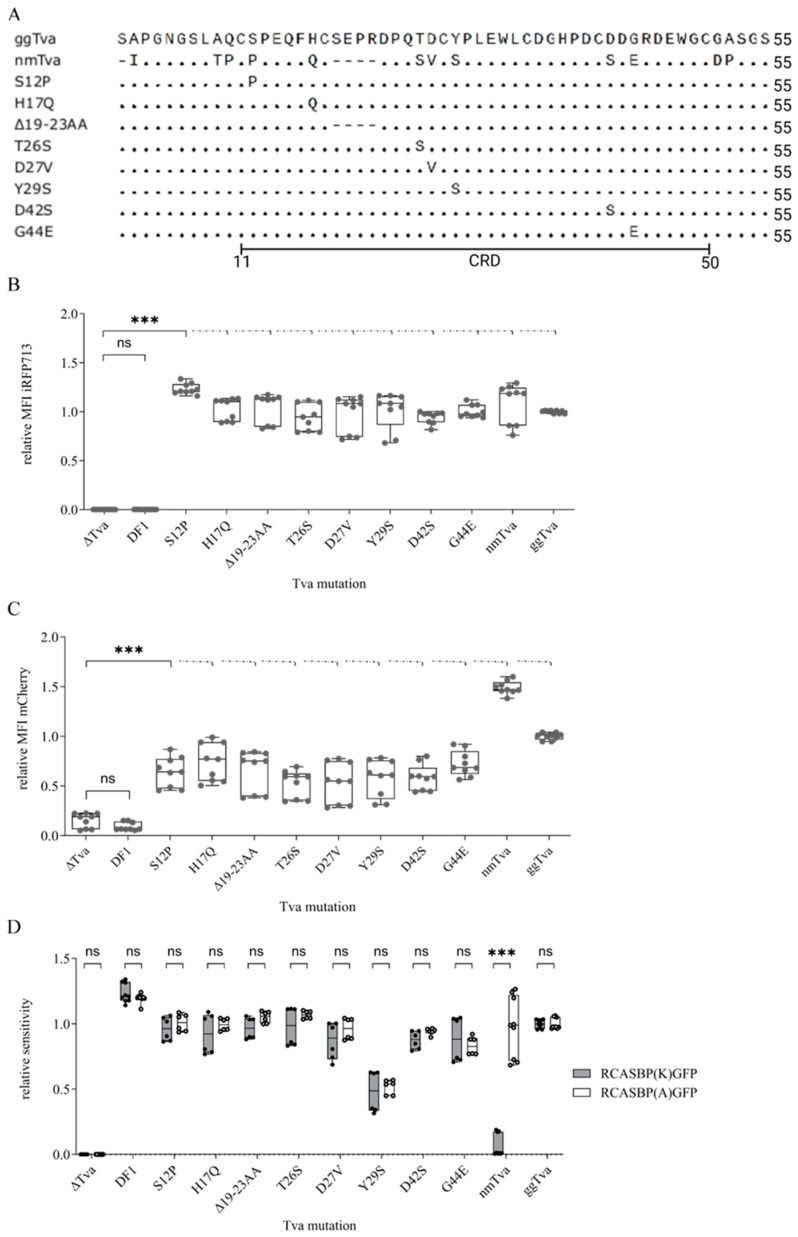
Single amino acid residue mutations and four amino acid residue deletion in CRD of *tva* receptors. (**A**) The *tva* sequence alignment of the 10 *tva* receptor variants. The sequence of gg*tva* and nm*tva* are displayed on the first two lines followed by sequences of eight mutant *tva* receptors: S12P, H17Q, Δ19-23AA, T26S, D27V, Y29S, D42S, and G44E. (**B**) The efficiency of receptor transduction, as determined by iRFP713 MFI normalized to the MFI of gg*tva*; Δ*tva* and DF1 were used as negative controls. (**C**) The relative expression of *tva* receptors fused with mCherry referred to gg*tva* expression. Δ*tva* and DF1 were used as negative controls and gg*tva* served as a positive control. (**D**) The infection assay of RCASBP(K)GFP and RCASBP(A)GFP recombinant viruses. Δ*tva* was the negative control, and DF1 and gg*tva* were the positive controls. The fractions of GFP-positive cells were normalized to the gg*tva*; Δ*tva* were used as negative control; DF1 and gg*tva* served as positive controls. Data are shown as means ± standard errors of three independent experiments with three biological replicates, ns, not significant, *** *p* < 0.001. Statistical analysis was performed using a two-way ANOVA test. ns: non-significant.

The CRD is involved in ALV-A entry, as evidenced by reduced virus infection in *tva* variants containing cysteine substitutions that lead to collapse of the CRD tertiary structure [26]. In addition, CRD contains the critical W48, which has been shown to play a key role in ALV-A binding and entry [19]. Our previous results indicate the importance of CRD also for ALV-K entry, as it overlaps with its tested proximal segment, which showed the biggest impact on the susceptibility to ALV-K infection. After aligning gg*tva* and nm*tva*, we found that the CRD contains seven different amino acid residue changes and a deletion of four amino acids (Figure 3A). To assess the significance of each mutation on mediating subgroup K entry, eight vectors, each containing the specific mutant, (FuTraP-gg*tva*-S12P, H17Q, T26S, D27V, Y29S, D42S, G44E, Δ19-23AA) were generated. Using these vectors, we transduced Δ*tva* cells, sorted them for iRFP713 positivity, and measured the mCherry expression. High iRFP713 MFI confirmed enrichment of the transduced population (Figure 3B) while expression of *tva* was verified comparing the mCherry expression (Figure 3C) among all constructs. The mCherry MFI of *tva* variants did not differ by more than two-fold from gg*tva* or nm*tva* receptors, assuring comparable expression levels.

To address whether these mutations influence the cellular entry of ALV subgroups A or K, the cells ectopically expressing mutant *tva* variants were infected with RCASBP(A)GFP or RCASBP(K)GFP viruses. The sensitivity of cells expressing individual receptors was determined as a fraction of GFP-positive cells and was normalized to the sensitivity of FuTraP-gg*tva* for the respective virus. Intriguingly, the infectivity of the viruses was not significantly altered by any of the mutations in the CRD (Figure 3D). Thus, our data suggest that no single polymorphic amino acid alone has a strong effect on *tva* receptor function and that the combination of multiple polymorphic amino acids throughout the CRD region is important for guineafowl resistance to subgroup K.

### 3.4. Tryptophan 33 in tva CRD Is Critical for ALV-K Binding and Infection

The above-described results show that the seven polymorphic amino acid residues in CRD of the Guineafowl *tva* do not confer the resistance to ALV-K individually and, therefore, cannot be used for the simple mutagenesis towards the chicken anti-virus resistance. In order to define any amino acid critical for ALV-K entry, we focused on tryptophan residues, because previous studies pointed to the indispensability of W48 in the *tva* for subgroup A virus binding and entry [19,27]. Furthermore, spatially prominent tryptophan residues have been shown critical also in different virus receptors, e.g., W38 in the Tvj receptor for subgroup J of ALV [28]. Therefore, we decided to focus on the tryptophan residues localized within the CRD region of *tva*, W33, and W48, and explore their importance in subgroup K binding and virus entry.

**Figure 4:**
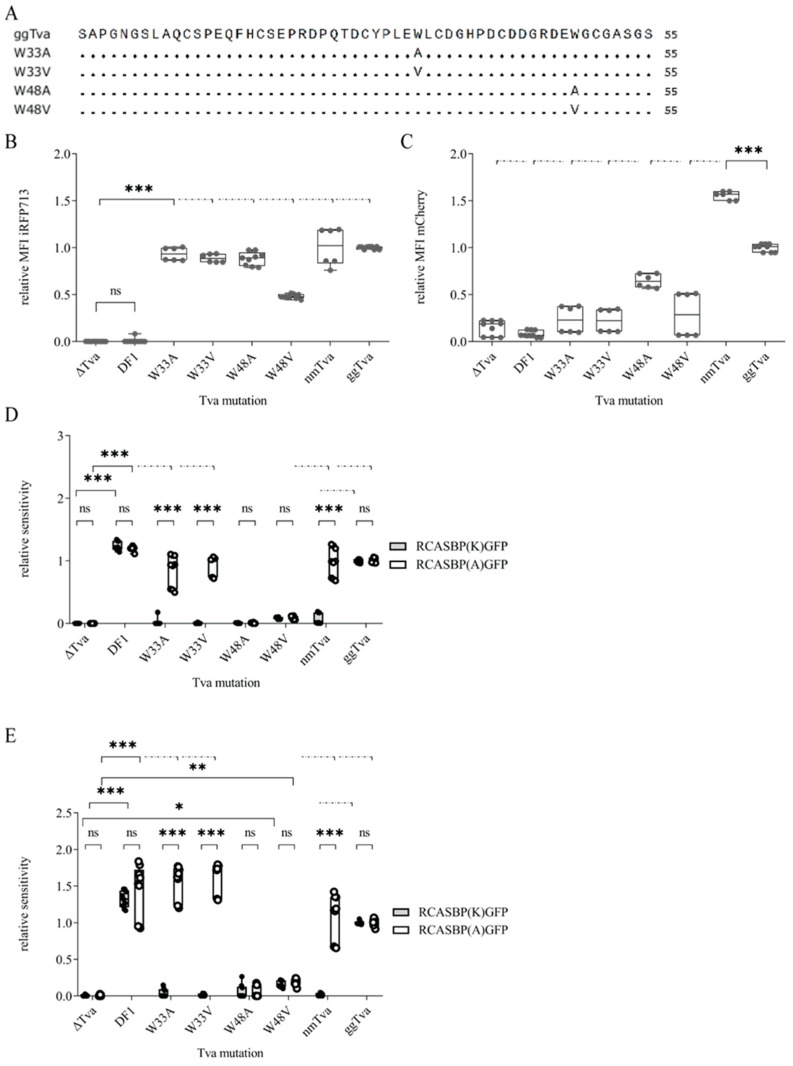
Mutant *tva* receptors with substitutions of tryptophans at positions 33 and 48. (**A**) The protein alignment of four mutant receptors. The sequences of gg*tva* and nm*tva* receptors shown on the first two lines, and four mutant receptors, labelled W33A, W33V, W48A, and W48V, are shown underneath. (**B**) The efficiency of transduction, as determined by iRFP713 MFI normalized to the MFI of gg*tva*; Δ*tva* and DF1 were used as negative controls. (**C**) The relative expression of *tva* receptors fused with mCherry referred to gg*tva* expression Δ*tva* and/or DF1—negative controls. (**D**) The infection assay of cells ectopically expressing mutant *tva* receptors infected with RCASBP(K)GFP and RCASBP(A)GFP recombinant viruses. (**E**) The spinoculation infection assay of mutant *tva* receptors with RCASBP(K)GFP and RCASBP(A)GFP viruses. (**D**,**E**) Δ*tva* and nm*tva* were used as the negative controls, and DF1 and gg*tva* as the positive controls. The GFP MFI of infected cells was normalized to the gg*tva*. Data are shown as means ± standard errors of three biological replicates, ns, not significant, * *p* < 0.05, ** *p* < 0.01, *** *p* < 0.001. Statistical analysis was performed using a two-way ANOVA test. ns: non-significant.

We generated tryptophan substitutions at positions 33 and 48 of chicken *tva*, by replacing it with alanine (W33A, W48A) or valine (W33V, W48V, Figure 4A) and ectopically expressed the respective *tva* variant in Δ*tva* cells. Cells successfully transduced with mutant *tva* receptors were enriched by sorting for iRFP713 positivity. With the exception of gg*tva*-W48V, transduction efficiency normalized to FuTraP-gg*tva* was comparable for all substitutions (Figure 4B). As assessed by mCherry fluorescence, all four *tva* receptor variants showed roughly the same expression level; however, the mCherry MFI median of mutant variants amounts to about one third of that of gg*tva* (Figure 4B). Despite the lower transduction efficiency even after enrichment (expressed as iRFP MFI) of FuTraP-W48V transduced cells, this variant expressed the receptor similarly to the other mutants (Figure 4C).

Next, we challenged cells expressing the above-mentioned *tva* variants with the RCASBP(A)GFP or RCASBP(K)GFP viruses. Sensitivity to infection was normalized to the gg*tva*. Both gg*tva*-W48 substitutions were resistant to infection with both subgroups. In striking contrast, the gg*tva*-W33 substitution mutants were sensitive to subgroup A infection, but resistant to subgroup K infection (Figure 4D). To strengthen our findings, we decided to enhance virus binding and entry by spinoculation, a method commonly used to enhance virus infection [29]. The sensitivity of W48 substitution mutants remained relatively unchanged, with a meager enhancement in infection for both subgroups. Strikingly, the W33 substitution mutants were sensitive to RCAS(A)GFP with 50% enhancement, while the resistance to the RCAS(K)GFP remained unchanged (Figure 4E). Thus, both infection and spinoculation assays demonstrated that while W48 is necessary to support infection by both subgroups, W33 is the critical amino acid residue exclusively for the entry of subgroup K.

**Figure 5:**
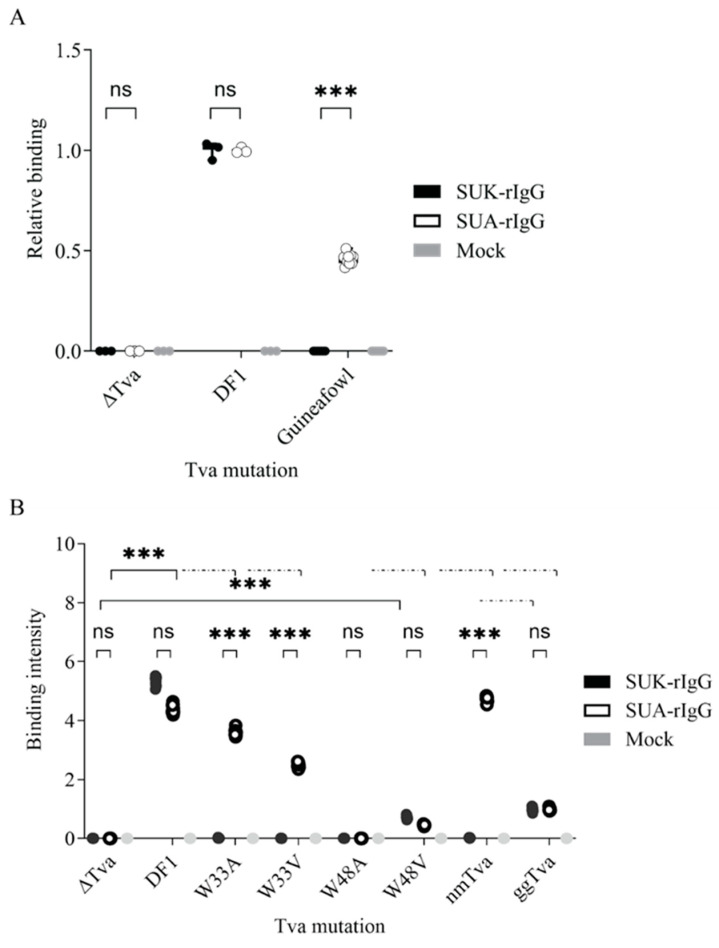
Immunoadhesin binding assay. The relative binding presented as MFI of Alexa Fluor 488-conjugated anti-rIgG detecting mutant *tva* receptors with substituted tryptophans at positions 33 and 48. (**A**) Binding of SUK-rIgG and SUA-rIgG to the ectopically expressed *tva* receptors with substituted tryptophans at positions 33 and 48. The binding intensity was normalized to the gg*tva*. (**B**) Binding of SUK-rIgG and SUA-rIgG to the endogenous wild-type *tva* of chicken the DF1 cells and guineafowl primary fibroblast. The binding intensity was normalized to the DF1. Δ*tva* was used as the negative control. Data are shown as means ± standard errors of three biological replicates, ns, not significant, *** *p* < 0.001. Statistical analysis was performed using a two-way ANOVA test. ns: non-significant.

Virus entry and infection rely on the envelope glycoprotein binding to its properly folded and surface-displayed receptor. To assess the binding of ALV-A or -K glycoproteins to respective *tva* variants, we employed an immunoadhesin, the soluble surface subunit (SU) of the envelope glycoproteins of subgroups A (SUA) and K (SUK) fused to the Fc region of rabbit IgG [30,31]. Live cells expressing the *tva* receptor variants were incubated with the cell supernatants containing the same amount of SUA-rIgG or SUK-rIgG proteins, and the binding of SUA and SUK was quantified using anti-IgG-Alexa Fluor 488 labeling. Both SUA-rIgG and SUK-rIgG bound efficiently to the endogenously expressed *tva* on chicken DF1 cells, but not to the Δ*tva* negative control (Figure 5A). In contrast, guineafowl embryo fibroblasts did not bind SUK-rIgG at all and SUA-rIgG binding was weaker than on DF-1 cells (Figure 5A) in accordance with much lower endogenous expression of *tva* in guineafowl (Figure 1A).

Next, we explored the binding of immunoadhesins derived from subgroups A and K to the cells ectopically expressing W33 and W48 *tva* substitution mutants. While SUA-rIgG immunoadhesin displayed high affinity to W33 mutants, SUK-rIgG was unable to bind to either of them (Figure 5B), suggesting that negligible ALV-K infection is due to weak binding and attachment of the virus to the receptor rather than blocking later stages of virus entry. As expected, neither of the immunoadhesin bound to gg*tva*-W48A, and only low-level binding of both SUA-rIgG and SUK-rIgG to gg*tva*-W48V was observed (Figure 5B). These results support the previous observations of the necessity of W48 for ALV-A entry, now extended to the ALV-K. Furthermore, our data suggest an additional independent crucial binding site for ALV-K, which does not affect ALV-A binding, clarifying the difference in epitopes recognized by each subgroup.

**Figure 6:**
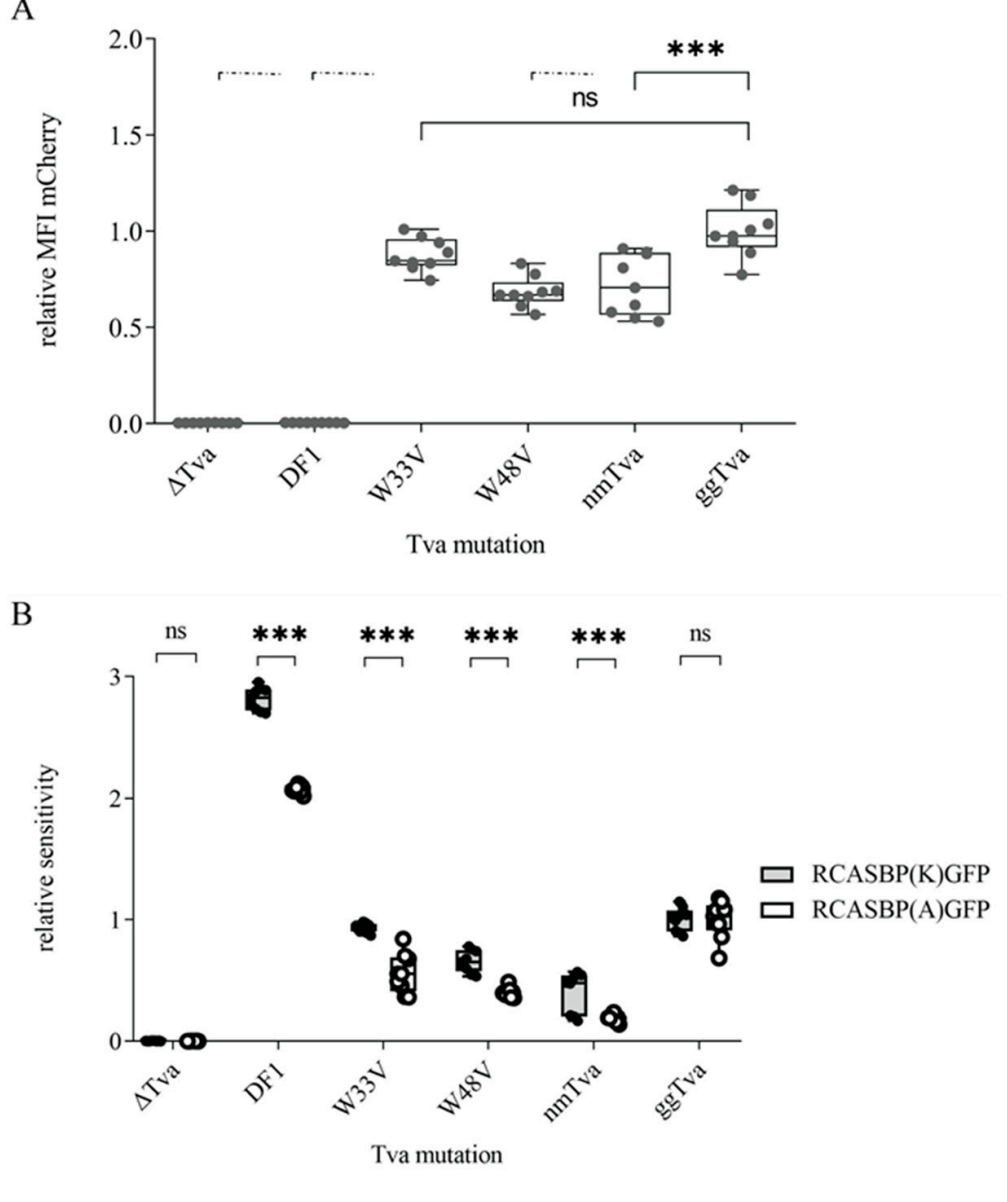
The effect of overexpressed *tva* receptor on virus entry. (**A**) The relative expression of ATG-initiated *tva* receptors fused with mCherry normalized to gg*tva* expression. Δ*tva* and DF1 were used as negative controls and gg*tva* served as a positive control. (**B**) The infection assay of cells overexpressing ATG-initiated mutated *tva* receptors infected with RCASBP(K)GFP and RCASBP(A)GFP recombinant viruses. Δ*tva* and nm*tva* were used as the negative controls, and DF1 and gg*tva* as the positive controls. The sensitivity to virus infection was normalized to the gg*tva*. Data are shown as means ± standard errors of three biological replicates, ns, not significant, *** *p* < 0.001. Statistical analysis was performed using a two-way ANOVA test. ns: non-significant.

Although the W33 substitutions did not cause any extensive structural changes leading to the removal of *tva* from the cell surface, as evidenced by the ability of SUA-rIgG to bind it, we could not exclude the possibility that the W48 substitutions altered the structure of *tva* so much that it was no longer present at the plasma membrane, because none of our immunoadhesins successfully bound to it. To verify the membrane display of the *tva* receptor with the substitution of the W48 mutation, we transiently overexpressed the *tva* receptor variants W33V and W48V from FuTraP vectors with the standard ATG initiation codon. All used constructs showed similar expression levels expressed as mCherry MFI (Figure 6A) and, therefore, were fit for infection by both subgroups. Cells expressing both *tva* variants exhibited sensitivity to both viruses that were comparable to nm*tva* and gg*tva* (Figure 6B), suggesting that the W48-mutated *tva* receptor can be exploited by ALV-A or -K subgroup for virus entry when overexpressed, confirming the proper display on the cell surface. Although we found some differences in sensitivity between different constructs to infection of subgroups A and K, indicating a low-affinity interaction with an abundantly expressed receptor, all of them were successfully infected by both subgroups. This again demonstrates the need to analyze *tva* mutants when expressed close to the endogenous level.

## 4. Discussion

In our previous research, we demonstrated that ALV-A and ALV-K share the *tva* as a cellular receptor [8]. However, their evolutionary distant Env glycoproteins and substantial difference in host range raise the possibility of distinct sites on the receptor recognized by the respective subgroup. Such a presumption implies that certain amino acid residues are critical for the entry of ALV-K, but not ALV-A, and vice versa. We took advantage of analyzing naturally occurring *tva* variants in galliform bird species resistant or sensitive to the ALV-K infection, an approach that has already been successfully applied in the search for amino acids critical for ALV-J cell attachment and entry [28]. Here, the key observation was of helmeted guineafowl being resistant to the ALV-K infection while sensitive to ALV-A [8]. Despite localizing the region necessary for productive infection of subgroup K viruses within the first 36 amino acids of the *tva* receptor, we failed to identify a single amino acid polymorphism crucial for virus binding and entry. Based on previous knowledge of the importance of the CRD and W48 for ALV-A entry, we focused on another tryptophan residue in this region, W33. We found W33 to be indispensable for ALV-K virus binding and entry to the host cells while retaining its ability to support subgroup A virus infection.

The fact that several different envelope glycoproteins share one receptor is not unique among retroviruses. ALV subgroups B, D, and E identically use the Tvb receptor, a tumor necrosis factor receptor-related protein [32,33,34]. As expected, competition for the same receptor leads to strong interference between the B and D subgroups. In contrast, subgroup E is not able to interfere with subgroup B or D superinfection. Reciprocally, cells preinfected with either B or D ALV subgroups become resistant to subgroup E superinfection. Adkins et al. [35] explained this pattern by two functionally distinct Tvb forms and non-overlapping binding sites for B, D, and E envelope glycoproteins. Similar to our case of subgroups A and K, different amino acid sequences critical for subgroup B and E binding have been identified previously [36,37]. The evolution of receptor sharing and what properties predispose the receptor to act as a hub for multiple retroviruses remains to be elucidated.

In order to identify amino acid residue critical for ALV-K interaction, we used a proven approach of ectopic expression of *tva* receptor variants in *tva*–deficient chicken DF1 cells. This effort, however, requires fine-tuned *tva* expression comparable to that of endogenous *tva*. We have shown previously that high levels of the mutant *tva* receptor may increase the overall avidity of the virus–receptor interaction to a level that allows ALV-A infection [13]. To avoid false sensitivity, we replaced the start codon ATG with TTG and decreased *tva* receptor expression driven from our constructs to a level normally found in chicken DF1 cells (Figure 1A). Using this strategy, we generated Δ*tva* cells ectopically expressing the guineafowl *tva* receptor that were completely resistant to ALV-K (Figure 1C,D). This cellular resistance to ALV-K recapitulated that of guineafowl and proved that ectopic *tva* expression, although higher than endogenous expression in guineafowl, did not confer any false sensitivity. Due to the low ectopic expression, however, it was not possible to directly visualize *tva* on the cell surface. Nevertheless, the display on the cell surface was demonstrated by virus infection when mutant *tva* variants were overexpressed (Figure 6B).

Notwithstanding the evidence of the importance of tryptophans 33 and 48 we provided in this study, the results revealed more complex interactions between ALV Env glycoproteins and the *tva* receptor. Firstly, despite identifying multiple amino acid sequence differences between helmeted guineafowl and chicken *tva* within the CRD, none of these polymorphic amino acids exerted a robust effect on ALV-K virus entry when substituted in chicken *tva* by their counterparts from guineafowl. The only exception is Y29S substitution, reducing the sensitivity of cells by 50% compared to chicken *tva* (Figure 3D). However, this reduction was observed for both viruses, suggesting a non-specific effect. It is plausible that a combination of specific amino acid substitutions is a necessary prerequisite, rendering the *tva* receptor unable to support a productive infection of subgroup K viruses. In a similar study [20], nine amino acids were selected from a comparison of chicken and human *tva* and only four of these were found to be important for ALV-K entry. However, only a combined substitution of all four amino acids abrogated the viral receptor function of chicken *tva* [20]. Unfortunately, the above mutants have not been tested for ALV-A entry and, therefore, we cannot state that these amino acids are specific for interaction with ALV-K. In our experiments, the ALV-A entry confirms that the *tva* variant in question acts as a virus receptor and thus its structure has not been grossly altered.

Secondly, the finding of critical importance of W33 does not explain the natural resistance of guineafowl to ALV-K because W33 remains intact in its *tva* receptor. Furthermore, northern bobwhite (*Colinus virginianus*) is resistant to the ALV-A infection while being susceptible to the ALV-K despite W48 of *tva* being substituted for serine [38]. These findings further support the need for multiple substitutions and the contextual and additive effect of specific amino acid residues among respective *tva* variants. Therefore, more studies regarding naturally occurring *tva* variants, and possibly different strains of ALV-A and -K viruses are necessary in the future to secure enough information about the virus–host arms race.

Overall, this study identified W33 as a critical residue for ALV-K binding and cell entry. Its substitutions W33A and W33V in the chicken *tva* receptor confer resistance to ALV-K and point to the possibility of creating ALV-K-resistant lines of domestic chicken by simple gene editing, as previously demonstrated for ALV-J and its receptor chNHE1 [12]. Here, however, it should be kept in mind that editing a single amino acid may not confer stable resistance or prevent selection of adapted virus variants [39]. Most probably, a more complex modification in the vicinity of the critical amino acid represents a stable solution and, therefore, future studies should focus on the vicinity of W33 and finding other target sites with similar or additive effects on the interaction with ALV-K.

## Figures and Tables

**Table 1:** PCR primers used for *tva* amplification, segment swapping, and mutagenesis.

NO.	Sequence 5′-3′
gg/nm*tva*-TTG-FW	ATACCAATTGCCACCTTGGTGCGGTTGTTGGAGC
gg/nm*tva*-TTG-RV	TGCGGATCAAGCTAGCAAGCCGTC
Segment_A-FW	CCGCTGCCCACCCCCA
Segment_A-RV	GTCGCAGAGCCACTCCAGC
Segment_B-FW	GCTGGAGTGGCTCTGCGAC
Segment_B-RV	CGGGGCTCCCGCTCG
Segment_C-FW	CGAGCGGGAGCCCCG
Segment_C-RV	GATAGCCCGCATAGTCAGGAACATC
gg*tva*-S12P-FW	ACTGCTCGGGCGGGCACTGCG
gg*tva*-S12P-RV	CGCAGTGCCCGCCCGAGCAGT
gg*tva*-H17Q-RV	GCTCCGAACACTGGAACTGCTCGG
gg*tva*-H17Q-FW	CCGAGCAGTTCCAGTGTTCGGAGC
gg*tva*-Δ19-23aa-FW	GCGGGTAGCAGTCGGTTTGGGGATCACAGTG-GAACTGCTCGGGTGAG
gg*tva*-Δ19-23aa-RV	CTCACCCGAGCAGTTCCACTGTGATCCCCAAAC-CGACTGCTACCCGC
gg*tva*-T26S-FW	GATCCCCAATCCGACTGCTACCCG
gg*tva*-T26S-RV	CGGGTAGCAGTCGGATTGGGGATC
gg*tva*-D27V-FW	CAGCGGGTAGCAGACGGTTTGGGG
gg*tva*-D27V-RV	CCCCAAACCGTCTGCTACCCGCTG
gg*tva*-Y29S-FW	CTCCAGCGGGGAGCAGTCGGTTTG
gg*tva*-Y29S-RV	CAAACCGACTGCTCCCCGCTGGAG
gg*tva*-D42S-FW	GTCCCGTCCATCGCTGCAGTCGG
gg*tva*-D42S-RV	CCGACTGCAGCGATGGACGGGAC
gg*tva*-G44E-FW	CCACTCGTCCCTTTCATCGTCGCA
gg*tva*-G44E-RV	TGCGACGATGAAAGGGACGAGTGG
gg*tva*-W48A-FW	ACGGGACGAGGCTtGGCTGCGGAGCGAGCGGG
gg*tva*-W48A-RV	GTCGCAGAGAGCCTCCAGCGGGTAGCAGTC
gg*tva*-W48V-FW	ACGGGACGAGGTTGGCTGCGGAGCGAGCGGG
gg*tva*-W48V-RV	CTCCGCAGCCAACCTCGTCCCGTCCATCGTCGC
gg*tva*-W33A-FW	CCCGCTGGAGGCTCTCTGCGACGGGCATCC
gg*tva*-W33A-RV	TCGCAGAGAGCCTCCAGCGGGTAGCAGTC
gg*tva*-W33V-FW	CCCGCTGGAGGTTCTCTGCGACGGGCATCC
gg*tva*-W33V-RV	GTCGCAGAGAACcCTCCAGCGGGTAGCAGTC

## Data Availability

Data are contained within the article.
